# Supplementary material for: Magnetic Resonance Imaging Liver Segmentation Protocol Enables More Consistent and Robust Annotations, Paving the Way for Advanced Computer-Assisted Analysis
Source: Diagnostics (Basel). 2024 Dec 11;14(24):2785. doi: 10.3390/diagnostics14242785 (PMC11726866; doi:10.3390/diagnostics14242785)
Supplement: Supplementary file 1 [file diagnostics-14-02785-s001.zip › diagnostics-3308980-supplementary.pdf]

Supplementary Materials:

Supplementary Table S1 presents the per-slice analysis using a Wilcoxon signed-rank test, treating each slice as independent. The per-slice inter-reader agreement evaluation exhibited a DSC enhancement after protocol implementation from 0.885 ± 0.208 to 0.924 ± 0.134 on T2wi and from 0.918 ± 0.145 to 0.925 ± 0.125 on T1wi, both with a statistically significant difference (p<0.001 on T2wi and T1wi). The HD reduced after protocol implementation from 8.73 ± 15.81 to 7.22 ± 13.45 on T2wi and from 6.26 ± 11.48 to 5.70 ± 9.80 on T1wi, both with a statistically significant difference (p<0.001 and p=0.035 on T2wi and T1wi, respectively).

Supplementary Table S1. Per-slice comparisons of metrics before and after protocol.

	Before protocol (± SD)	After protocol (± SD)	Wilcoxon signed-rank test p-value
**T2wi**	DSC = 0.885 ±0.208	DSC = 0.924 ±0.134	**<0.001**
	HD = 8.73 ±15.81	HD = 7.22 ±13.45	**<0.001**
**T1wi**	DSC = 0.918 ±0.14	DSC = 0.925 ±0.12	**<0.001**
	HD = 6.26 ±11.48	HD = 5.70 ±9.80	**0.035**

Abbreviations: SD: standard deviation, wi: weighted imaging, DSC: Dice Similarity Coefficient, HD: Hausdorff Distance.

Supplementary Table S2 presents the per-slice analysis using the Wilcoxon signed-rank test, applied in the subgroup analysis for patients with and without cirrhosis based on the assumption that slices are independent. The protocol implementation showed improvement of the DSC for the metrics measured on T2wi for patients with and without cirrhosis and on T1wi for patient with cirrhosis, with a statistically significant difference (p<0.001). However, no significant improvement in DSC was observed on T1wi for patients without cirrhosis (p=0.82).

Supplementary Table S2. Comparisons of metrics in the subgroup analysis for patients with and without cirrhosis.

	Weights	Per-slice analysis (p-value)	DSC improvement
Cirrhosis	T2wi	<0.001	Yes
Without cirrhosis	T2wi	<0.001	Yes
Cirrhosis	T1wi	<0.001	Yes
Without cirrhosis	T1wi	0.82	No

Abbreviations: DSC: Dice Similarity Coefficient, wi: weighted imaging.
